# Immune Response in Traumatic Brain Injury

**DOI:** 10.1007/s11910-024-01382-7

**Published:** 2024-10-29

**Authors:** Eder Cáceres, Juan Camilo Olivella, Mario Di Napoli, Ahmed S. Raihane, Afshin A. Divani

**Affiliations:** 1 Unisabana Center for Translational Science, Universidad de La Sabana, Chía, Colombia; 2 School of Medicine, Universidad de La Sabana, Chía, Colombia; 3 Bioscience PhD. School of Engineering, Universidad de La Sabana, Chía, Colombia; 4 Neurological Service, SS Annunziata Hospital, Sulmona, L’Aquila Italy; 5 School of Medicine, University of New Mexico, Albuquerque, NM USA; 6 Department of Neurology, University of New Mexico Health Science Center, Albuquerque, NM USA

**Keywords:** Trauma, Brain injury, Inflammation, Inflammasome, Immune response, Innate immunity, Adaptive immunity, Autonomic system, Vagus nerve, Cholinergic pathway

## Abstract

**Purpose of Review:**

This review aims to comprehensively examine the immune response following traumatic brain injury (TBI) and how its disruption can impact healing and recovery.

**Recent Findings:**

The immune response is now considered a key element in the pathophysiology of TBI, with consequences far beyond the acute phase after injury. A delicate equilibrium is crucial for a healthy recovery. When this equilibrium is disrupted, chronic inflammation and immune imbalance can lead to detrimental effects on survival and disability.

**Summary:**

Globally, traumatic brain injury (TBI) imposes a substantial burden in terms of both years of life lost and years lived with disability. Although its epidemiology exhibits dynamic trends over time and across regions, TBI disproportionally affects the younger populations, posing psychosocial and financial challenge for communities and families. Following the initial trauma, the primary injury is succeeded by an inflammatory response, primarily orchestrated by the innate immune system. The inflammasome plays a pivotal role during this stage, catalyzing both programmed cell death pathways and the up-regulation of inflammatory cytokines and transcription factors. These events trigger the activation and differentiation of microglia, thereby intensifying the inflammatory response to a systemic level and facilitating the migration of immune cells and edema. This inflammatory response, initially originated in the brain, is monitored by our autonomic nervous system. Through the vagus nerve and adrenergic and cholinergic receptors in various peripheral lymphoid organs and immune cells, bidirectional communication and regulation between the immune and nervous systems is established.

## Introduction

Traumatic brain injury (TBI) is a leading cause of mortality and disability worldwide, involving complex pathophysiological pathways. This chapter will discuss several of these mechanisms. Clinical outcomes following TBI are influenced by multiple factors, including the type, location, and severity of intracranial lesions, as well as extracranial injuries and systemic conditions like hypoxia or hypotension [1, 2]. Despite advances in multimodality monitoring and precision medicine, TBI remains a challenging and poorly understood condition, characterized by significant intra- and inter-individual variability. Current monitoring tools utilize advanced technology to assess the metabolic and inflammatory response to the injury, guiding therapy and interventions. However, the interpretation and standardization of these tools remain difficult, and their clinical utility is often debated. Additionally, their availability is limited, and in some cases, they are used primarily for research purposes [3, 4]. TBI injuries can be divided into primary and secondary stages. Primary injury results from direct, blunt, or penetrating impacts, acceleration/deceleration forces, or blast injuries, leading to focal intracranial hemorrhage, epidural or subdural hematoma, brain contusion, and/or diffuse axonal damage [3]. Secondary injury is initially mediated by the innate immune system, which triggers the inflammatory response to the structural and homeostatic disruption of tissues. This response can be either beneficial or detrimental to the patient's outcome depending on its intensity, duration, and modulation. The mechanisms of the secondary injury are multifaceted and closely related to the immune response; some of these mechanisms include oxidative stress, blood–brain barrier (BBB) disruption, excitotoxicity, edema, ischemia, and cell death [5, 6].

Later, during the chronic phase after trauma, patients frequently remain at higher risk of long-lasting processes like chronic inflammation, neurodegeneration, cognitive impairment, and metabolic and cardiovascular diseases [7–9]. While these mechanisms may seem straightforward, they involve multiple complex processes that have been the focus of extensive research in both animal and clinical models, limiting a comprehensive understanding of the entire picture. Additional challenges stem from factors such as the age, gender, and genetic background of the species used. No single model can fully encompass all the primary and secondary injuries observed in clinical settings post-TBI [10, 11]. This introduction highlights the complexity of TBI and the importance of understanding the immune response secondary to the injury. It also underscores that many questions remain unanswered. This review will focus on the immune mechanisms involved in TBI, their relationship with the inflammasome, and their systemic interactions.

## Innate and Adaptive Immunity

The initial structural damage associated with TBI is caused by external forces impacting the head. The type of injury is heterogeneous, depending on the specific mechanism of trauma: blunt injury, penetrating injury, acceleration/deceleration, coup/countercoup, diffuse axonal injury, and skull fracture. Although variable in nature, these types of primary injury share a common element: the disruption of local tissues at the macroscopic and cellular levels, which ensues in physiological and homeostatic disturbances that would affect the clinical neurological condition of the patient at a degree related to the type, severity, and extent of the injury [12, 13]. In addition, the local structural damage precipitates the release of various cell membrane fragments and intracellular content into the extracellular environment and systemic circulation, triggering an immune response initially driven by the innate immunity and posteriorly involving the adaptive immune system. Understanding the roles of innate and adaptive immunity is crucial in elucidating the pathways implicated in TBI.

### Innate Immunity

Shear forces during TBI disrupt cell membranes and vascular structures, releasing intracellular and membrane components. Some of these components act as damage-associated molecular patterns (DAMPs), which are recognized by inflammatory and non-inflammatory cells through membrane and soluble receptors known as Pattern Recognition Receptors (PRR) [14, 15].

### Damaged-Associated Molecular Patterns

DAMPs are endogenous molecules released from damaged cells and tissues that are recognized by PRRs as indicators of cellular stress or tissue injury, triggering an inflammatory response [17]. DAMPs include intracellular and extracellular matrix molecules such as High Mobility Group Box 1 (HMGB1), Heat Shock Proteins (HSPs), S100 proteins, hyaluronic acid, mitochondrial peptides, and mitochondrial DNA, among others [16, 18, 19]. Depending on the intracellular signaling pathways activated, DAMPs can induce different types of cellular responses. For example, HSPs and other DAMPs can act as ligands for multiple PRRs, playing dual roles as immunomodulators or pro-inflammatory agents. HSP70, for instance, can modulate TNF-α, IL-6, and nitric oxide production; overexpression of HSP70 in animal models has been shown to reduce brain lesion size, hemorrhage, and metalloproteinase expression [20, 21]. DAMPs can be recognized in the brain by specific PRRs present in different cell types, including neurons, astrocytes, and microglia [22, 23].

### Pattern Recognition Receptors

PRRs are a key element of the innate immune response, initially recognized for their ability to bind specific pathogen-associated molecular patterns expressed by microorganisms, constituting the first line of defense against infectious agents [24]. However, PRRs also play a role in sterile inflammation by responding to endogenous stimuli known as DAMPs or alarmins. Types of PRRs include Toll-like receptors (TLR), Nod-like receptors (NLR), AIM2-like receptors (ALR), C-type lectin receptors, and RIG-like receptors. Some of these receptors are expressed on the cellular membrane, while others are soluble and expressed intracellularly [24, 25].

### Toll-Like Receptors

TLRs are part of the Toll-interleukin-1 receptor family and initiate dimerization and recruitment of adapter proteins such as MyD88 and TRIF, which activate signaling pathways leading to the translocation of NFkB, and the subsequent production of inflammatory mediators as well as other processes involved in cell survival, proliferation, and differentiation [24, 26]. NF-κB (nuclear factor kappa-light-chain-enhancer of activated B cells) is a crucial transcription factor orchestrating the inflammatory response in the CNS. Its activation is a key event in neuroinflammation following TBI; NF-κB is usually held inactive in the cytoplasm by inhibitory proteins known as IκBs. Upon stimulation by signals such as DAMPs, cytokines, and oxidative stress, IκB proteins are phosphorylated and degraded. This degradation activates NF-κB, allowing it to translocate to the nucleus. In the nucleus, NF-κB binds to specific DNA sequences, promoting the transcription of genes involved in the inflammatory response [27].

Up to 10 types of TLRs have been identified (TLR1- 10), expressed either intracellularly (TLR3, TLR7, TLR8, TLR9) or as transmembrane receptors (all others). TLR4, for example, is expressed in the membrane of microglia, astrocytes, neurons, and endothelial cells and can recognize endogenous proteins like HMGB1, HSPs, and low-density lipoprotein [28]. This recognition initiates signal transduction pathways via MyD88 and TRIF, which lead to the activation of the transcription factor NF- κB and mitogen-activated protein kinases (MAPKs). NF- κB regulates the expression of inflammatory cytokines (IL-1, IL-6, TNF-α, IL-18), chemokines, intracellular (ICAM-1), vascular (VCAM-1), and endothelial (ECAM-1) adhesion molecules, and type I IFNs. NK-kB not only induces the expression of inflammatory cytokines, chemokines, and adhesion factors but also regulates pathways concerning cell survival, maturation, and differentiation of inflammatory cells such as microglia and astrocytes [29, 30].

Synergistic cooperation between PRRs has been established; for example, IL-1β and IL-18 are initially translated as their precursors, pro-IL-1β and pro-IL-18. A second signal is necessary to activate a different PRR, NLR,. NLRP3, known for forming the inflammasome, subsequently activates caspase-1, which cleaves pro-IL-1β and pro-IL-18 into their active forms [31–33].

### Nod-Like Receptors

NLRs are a family of PRRs expressed in the cytosol and constantly assess the intracellular environment for signs of injury or metabolic stress. NLRs share a common structure consisting of a leucine-rich repeat (LRR), a nucleotide-binding oligomerization domain (NOD), and a variable N-terminal effector region that can be the caspase activation and recruitment domain (CARD), pyrin domain (PYD), acidic domain or baculovirus inhibitor repeats (BIRs) [34]. Depending on the effector region, there are several NLR families: NLRA, NLRB, NLRC, and NLRP. This review will keep particular interest in NLRP1, NLRP3, and NLRP4, which play a prominent role in activating caspase-1 in response to noxious stimuli. Caspase-1 catalyzes the cleavage of IL-1β and IL-18 into their active forms [35]. The assembly of the NLR protein, the adaptor ASC (apoptosis-associated speck-like protein containing a C-terminal CARD), and the pro-caspase-1 form a protein complex known as the inflammasome. Inflammasomes are macromolecular complexes that activate caspases and induce the production of cytokines. Once activated, NLRs oligomerize with ASC to activate inflammatory caspases, such as caspase-1, which cleave and activate proinflammatory cytokines [36].

This process leads to the cleavage of pro-caspase-1 into its active form, caspase-1, which subsequently induces the cleavage of cytokine precursors IL-1β and IL-18 into their active forms. Caspase-1 also processes the pore-forming protein gasdermin D (GSDMD), whose activity induces a form of cell death known as pyroptosis. Together, the production of inflammatory cytokines and the release of intracellular components after cell death contribute to the sterile immune responses following brain injury [37, 38].

Not all inflammasomes require ASC for oligomerization. For instance, NLRP1 and NLRC4 can activate caspase-1 independently, although this activation is generally weaker compared to ASC-dependent pathways [39]. Inflammasomes also induce cell death pathways such as necroptosis and pyroptosis, which can enhance antigenic stimulation by releasing cellular components [40].

Inflammasomes can be assembled in multiple cell populations in the central nervous system (CNS), such as microglia, macrophages, astrocytes, and neurons. Evidence suggests that inflammasome expression can vary between different types of cells. For example, microglia seem to express NLRP3 inflammasome, but not NLRP1 [41], while NLRP1 has been found more frequently in neurons [42].

Several molecules have been proposed as biomarkers for injury severity, response to treatment, and predictors of outcomes after head trauma, including glial acidic fibrillary protein (GFAP) [43], neuron-specific enolase (NSE), and S100B [44]. In this context, inflammasomes have been evaluated for their diagnosis and prognostic potential following TBI [47] and have been proposed as therapeutic targets [45].

Inflammasome proteins such as NLRP1, ASC, and caspase-1 have been detected in the cerebrospinal fluid (CSF) of patients with moderate to severe TBI. Their concentration directly correlates with unfavorable outcomes and the systemic response after trauma [46]. In a study measuring levels of ASC, IL-18, and caspase-1 in serum and CSF of patients with TBI and control donors without TBI, higher concentrations were observed in the TBI group, suggesting a potential diagnostic and prognostic role [47]. The two main proteins detected in CSF samples were ASC and IL-18, while caspase-1 was not detected. These findings suggest that ASC may be a more reliable biomarker than IL-18. Additionally, ASC levels were associated with unfavorable outcomes determined by the Glasgow Outcome Scale-Extended (GOSE), positioning it as a candidate for a prognostic biomarker in TBI [47]. Furthermore, a parallel has been drawn between inflammasome activation in TBI, chronic traumatic encephalopathy, and Alzheimer´s disease as a common pathophysiological pathway [48–50].

### RIG-Like Receptors

Retinoic acid-inducible gene-1 (RIG-1), melanoma differentiation-associated gene-5 (MDA5), and laboratory of genetics and physiology-2 (LGP2) are part of the cytosolic PRR family RIG-like receptors (RLR) which are primarily involved in recognizing viral RNA and inducing the transcription of type 1 IFNs (IFN- αand IFN-β [54]. However, type I IFN production by astrocytes has also been documented [50]. In spinal cord injury and stretch injury models, astrocytes show elevated levels of intermediates involved in RLR activation, including IFN-α and IFN-β. Additionally, markers of astrocyte activation, such as GFAP and vimentin, might be related to reactive astrogliosis after injury [51].

The release of nucleic acid fragments associated with injury may contribute to RLR sterile activation within the CNS [52]. An increase in RIG-1 and type I IFN expression has also been observed in ischemic stroke models involving middle cerebral artery occlusion [54]. Whether the role of type I IFNs is beneficial or detrimental role requires further investigation. On one side, it could contribute to hypoxia-induced neuroinflammation [53]; on the other, it may limit the infiltration of monocytes and neutrophils into the brain [54].

### AIM-2-Like Receptors

Absent in melanoma-2 receptors (ALR) are cytosolic receptors that can recognize viral DNA, but they also play a role in responding to danger signals after CNS injury. This initial response appears to be driven by the release of endogenous double-stranded DNA into the cytosol of microglia [55]. ALR activation induces the expression of type I IFNs by activating transcription factors NF- κB and interferon regulatory factor 3 (IRF3) [56]. Additionally, ALR activation triggers the assembly of the AIM2 inflammasome, which is involved in sterile inflammation caused by endogenous DNA. This leads to the cleavage of caspase-1, followed by maturation of IL-1β and GSDMD, thereby amplifying inflammation and initiating pyroptosis [57–59]. AIM2 inflammasome activation is associated with BBB disruption, which facilitates the systemic release of inflammatory markers. AIM2 may also contribute to cardiac dysfunction and acute lung injury after TBI, potentially playing a role in multiorgan dysfunction, a common cause of death and disability following TBI [58, 60, 61].

### C-Type Lectin Receptor

C-type lectin receptors (CLR) are a family of transmembrane receptors with a carbohydrate-binding domain within their structure [62, 63]. Increased expression of CLRs has been documented after TBI, correlating with the severity of trauma and mortality rates [64]. CLRs, specifically mannose-binding lectin (MBL), recognize pathogenic carbohydrates, and its activation has been demonstrated to induce BBB disruption, neutrophil infiltration, and demyelination several days after TBI [65]. MBL is associated with circulating serine proteases (MASP-1 and MASP-2); the activation of the MBL/MASP complex is the initial step in the lectin complement pathway, which can activate C3 convertase and C4 cleavage independently of the classical pathway. The classical pathway is initiated by C1 complex activation by myelin and neuronal debris in the context of brain injury [65, 67, 68]. MBL has been proposed as a therapeutic target after TBI, as its inhibition can increase neurogenesis, reduce circulating C4b levels, and improve sensorimotor function [66]. Another group of PRRs includes scavenger receptors (SRs). SRs are a family of cell surface receptors that bind pathogen-associated ligands and endogenous patterns like oxidized LDL and amyloid β [69]; CD36 is a member of the SR family that can convert endogenous soluble ligands such as amyloid β, into amyloid oligomers, which in turn, activate the NLRP3 inflammasome and IL-1β, perpetuating inflammation [70].

## Innate Immune Response After TBI

Following injury and exposure to damage-associated molecular patterns (DAMPs), effector cells in the brain, including microglia, astrocytes, and neurons, become activated. These cells engage in pathways that stimulate transcription factors, release cytokines, chemokines, and adhesion molecules, and initiate processes related to cell death, proliferation, and differentiation. This initial response triggers the release of various inflammatory mediators, which further activate and differentiate microglia and astrocytes. This process facilitates the infiltration of neutrophils, monocytes, and macrophages from the peripheral circulation [71].

Depending on factors like the microenvironment, type of injury, and epigenetics [72], microglia and macrophages can differentiate into a spectrum of pro-inflammatory (M1) and anti-inflammatory (M2) phenotypes. These phenotypes direct inflammation and tissue repair [73]. Microglia in the pro-inflammatory spectrum (M1-like) primarily release inflammatory cytokines such as IL-1β, IL-6, IL-12, TNF-α, and chemokines like CCL2 and CXCL9 [74]. Conversely, M2-like differentiated microglia exhibit an anti-inflammatory profile, limiting inflammation and promoting tissue repair by releasing IL-4, IL-10, and IL-13, and transforming growth factor-β [75, 76]. Microglia play a critical role in maintaining or disrupting neuronal homeostasis in the subacute and chronic phases following TBI through differentiation and cytokine and chemokine production [77]. Initially, microglia-mediated inflammation helps resolve the injury by clearing debris and promoting tissue repair. However, if this inflammatory state persists, it can lead to structural changes and neurodegeneration by inhibiting axonal growth, synaptic formation, and remyelination [76, 78].

## Role of Neutrophils and Complement System

Neutrophils also contribute to the innate immune response in TBI. In response to inflammatory cytokines, TNF-α, and IL-1β, various epithelial tissues produce chemokines (e.g., CXCL1, CXCL2, CXCL3) that drive neutrophil migration into the CNS [79]. Once in circulation, neutrophils are attracted to the endothelium, binding to ICAM-1 and ICAM-2, and subsequently migrate to the injury site, where they contribute to phagocytosis and tissue debris clearance, aided by complement protein C3d opsonization [80]. However, BBB disruption and peripheral immune cell infiltration may create a feedback loop reinforcing secondary injury [81].

The complement system, another important player in the innate immune response to TBI, involves circulating complement proteins primarily produced by the liver, which are unlikely to cross the BBB [82]. Complement protein expression has been documented in astrocytes and microglia, explaining the elevated levels of complement components like C1q, C3b, C3d, and C5b-9 (membrane attack complex, MAC) found in areas of penumbra and contusion after TBI. This suggests that complement activation may be triggered by myelin or neuronal debris [67]. MAC is a transmembrane protein complex that can form pores in cell membranes, causing cell lysis and promoting microglia activation, neuronal apoptosis, and axonal loss [68], thereby contributing to the innate immune response after TBI.

**Fig. 1:**
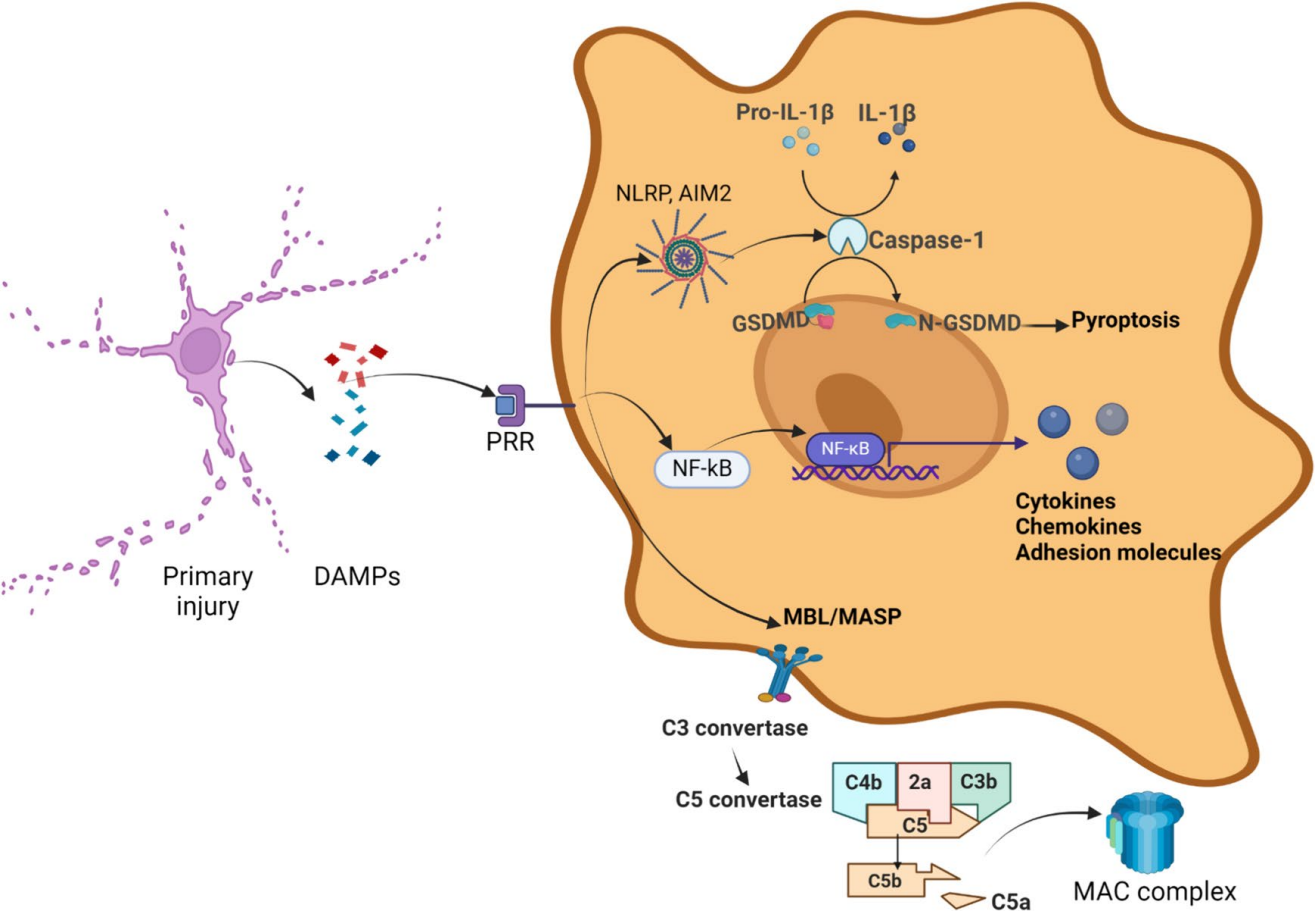
Innate immunity. The primary insult from external forces leads to cellular disruption and the release of membrane and cytosolic components that function as damage-associated molecular patterns (DAMPs). These DAMPs include membrane fragments, heat shock proteins (HSPs), high mobility group box 1 (HMGB1), and mitochondrial DNA, among others. DAMPs are recognized as ligands by various pattern recognition receptors (PRRs), such as toll-like receptors (TLRs), nucleotide-binding oligomerization domain-like receptors (NLRs), AIM2-like receptors (ALRs), C-type lectin receptors (CLRs), and scavenger receptors, which are linked to several intracellular pathways. ALRs and NLRs facilitate the assembly of the AIM2 and NLRP inflammasomes, which converge to activate caspase-1. Caspase-1, in turn, mediates the cleavage of pro-IL-1β into its active form, IL-1β, and gasdermin D (GSDMD) into its active form, N-GSDMD. GSDMD is an effector of the pyroptosis cell death pathway due to its ability to form pores in the cell membrane, leading to cell swelling and rupture. Additionally, IL-1β induces the expression of other cytokines such as IL-2 and IFN-γ and stimulates the activation and differentiation of T and B cells. TLRs initiate dimerization and recruit adapter proteins, such as MyD88 and TRIF, which activate signaling pathways and lead to the translocation of NF-κB. This process results in the production of cytokines, chemokines, and adhesion molecules, which facilitate the migration and infiltration of neutrophils and monocytes from the peripheral circulation. Another PRR family, CLRs—specifically mannose-binding lectin (MBL)—forms a complex with serine proteases, known as MASP (mannose-binding lectin-associated serine proteases), which activates the lectin complement pathway. This pathway leads to the formation of the membrane attack complex (MAC), which has a cytolytic effect by forming pores in the cell membrane. Acronyms: ALR: AIM2-like receptor; CLR: C-type lectin receptor; DAMP: Damage;associated molecular pattern; GSDMD: Gasdermin D; HMGB1: High mobility group box 1; HSP: Heat shock protein; MAC: Membrane attack complex; MBL: Mannose;binding lectin; MASP: Mannose;binding lectin-associated serine protease; NLR: Nucleotide-binding oligomerization domain-like receptor; PRR: Pattern recognition receptor; TLR: Toll-like receptor

TBI disrupts cellular and vascular components in the brain, triggering an immediate activation of innate immunity. This process involves resident cells and inflammatory mediators through the activation of PRRs, leading to the stimulation of transcription factors and altered expression of inflammatory molecules. These changes induce microglia and astrocytes differentiation and maturation, alongside the migration of inflammatory cells from the periphery (neutrophils, monocytes) and complement activation via the inflammasome and cellular components. These mechanisms play a crucial role in enhancing a diffuse and non-specific innate immune response. However, this initial response to injury also initiates a more antigen-specific or adaptive immune response mediated by T and B lymphocytes (Fig. 1).

### Adaptive Immunity

After the initial innate immune response, the adaptive immune system, mediated by B and T lymphocytes, begins to act. This system is tailored to recognize specific antigens encountered during the injury and retain memory of the insult for future responses [83].

The innate and adaptive immune systems do not function in isolation; instead, they interact dynamically to shape the overall immune response to TBI. For instance, innate immune cells such as microglia and macrophages present antigens to T cells, linking the innate and adaptive responses. The adaptive response consists of cellular and humoral components, carried out by T and B lymphocytes, respectively [84]. Following TBI, there is an increase in the leukocyte count in the peripheral circulation, likely related to the body's cortisol and catecholamine response. This facilitates the migration and activation of lymphocytes in the bone marrow and peripheral lymphoid organs, such as the spleen, lymph nodes, and gut-associated lymphoid tissue [85]. The adaptive immune response has two main components: cell-mediated and humoral responses.

### Cell-Mediated Adaptive Immune Response

The initial recruitment of T lymphocytes into the injured tissue is facilitated by the upregulation of cell adhesion molecules on vascular endothelium (such as E-selectin and ICAM-1) and chemokines (such as RANTES). These adhesion molecules and chemokines are present in the leptomeninges and choroid plexus, enabling the adaptive immune response to be involved in the brain [86, 87].

Reactive oxygen species (ROS) produced at the injury site can also drive infiltration by activated T lymphocytes [89]. Once at the injury site, T lymphocytes come into contact with antigen-presenting cells (APC), specifically microglia and perivascular macrophages [88]. Major histocompatibility complexes (MHC) I and II are upregulated in macrophages and microglia after TBI [90]. MHC molecules present antigen peptides to T cells: MHC class I is expressed on nearly all nucleated cells and presents endogenous peptides to the T cell receptor (TCR) on cytotoxic CD8 + T cells [91]. CD8 + T cells primarily control malignancies and intracellular infections but can also release pore-forming enzymes and induce apoptosis in neurons and glial cells following TBI [92]. MHC class II is expressed in APCs and presents antigens to the TCR on CD4 + T cells; CD4 + T cells, on the other hand, act by coordinating and regulating effector cells [93]. Various subsets of CD4 + lymphocytes exist, and their differentiation is influenced by cytokine signaling, transcription factors, and epigenetic factors [94].

The MHC II-antigen complex, along with costimulatory molecules like CD80 and CD86, generates an internal T cell response whose strength depends on factors such as antigen affinity, dose, the type of APC, and the cytokines present in the microenvironment [95, 96].

TCR activation triggers a complex network of internal signaling that leads to the differentiation and proliferation of naive CD4 + lymphocytes into five major CD4 + T helper cell (Th) types: Th1, Th2, Th17, Treg (T regulatory), and Tfh (follicular helper) [97, 98]. Each subset of T cells secretes specific cytokines, thereby regulating the direction and strength of the inflammatory response following TBI. Th1 and Th17 cells play a pro-inflammatory role, with Th1 cells producing IL-2, TNF-α, and IFN-γ, and Th17 cells secreting IL-17, IL-22, and IL-23. The predominance and lack of modulation of this response have been associated with white matter injury, neuronal damage, and demyelination [99–101].

In contrast, Th2 and Treg cells have more anti-inflammatory or modulatory functions. Th2 cells produce cytokines like IL-4, IL-5, IL-10, and IL-13, which promote tissue repair by activating macrophages that stimulate fibroblast proliferation and the removal of extracellular matrix and apoptotic cells [102]. Treg cells function by influencing other T cells, reducing brain infiltration by T cells, mitigating reactive astrogliosis, and attenuating IFN-γ gene expression [103, 104].

Treg cells also play a critical role in controlling autoimmunity and promoting peripheral tolerance to neural antigens [105]. Additionally, in the CNS, IL-4-associated macrophages promote oligodendrocyte differentiation and axonal growth [106, 107]. Typically, a proinflammatory response dominates the initial response after injury; however, failure to switch to a modulatory response or persistence of a proinflammatory state can be deleterious and delay healing and recovery [108–110]. Tfh cells are in the follicular zone of lymphoid tissue, where they assist in developing antigen-specific B-cell immunity. Tfh cells in the cervical lymph nodes are part of the communication pathways between the CNS and the immune system via the meningeal lymphatic system [111]. The meningeal lymphatic system may receive cerebrospinal fluid (CSF) from the surrounding subarachnoid space and brain interstitial fluid through the glymphatic system. This challenges the traditional view that lymphatic vessels are limited to connective tissue and that CSF drainage occurs exclusively via arachnoid projections and dural venous sinuses [112].

### Antigen-Specific Adaptive Immune Response

The effectors of the antigen-specific adaptive immune response are the T and B cells. B lymphocytes originate in the bone marrow and, after entering the circulation, eventually localize to the spleen and lymph nodes [113]. B cell activation requires binding the B cell receptor (BCR) to a specific antigen, either through direct recognition or presentation by T helper cells. There must be a costimulatory signal from B cell co-receptors CD19, CD21, and CD81. Once the B cell is activated, the antigen is internalized, processed into peptides, and presented on the cell surface via MHC II molecules. MHC II is recognized by CD4 + and CD8 + T cells. Additionally, T cells must display the CD40 ligand, which binds the CD40 receptor on the B cell surface as another co-stimulatory signal. After this activation, T helper cells produce cytokines such as IL-4, IL-5, and IL-6, stimulating B cells' proliferation and differentiation [114, 115].

The antigen-binding portion of the BCR is a cell surface immunoglobulin structurally similar to the antibodies that the B cell will ultimately produce. The BCR consists of two identical light chains, two identical heavy chains, and two proteins named Igα and Igβ (also known as CD79A and CD79B) connected via a transmembrane domain to the cytoplasmic tail. Igα and Igβ are essential for intracellular signaling after the BCR has recognized an epitope. These proteins contain immune-receptor tyrosine-based activation motifs (ITAMs), which, once phosphorylated, trigger downstream signaling via NF- κB, PI3K, and calcium mobilization to stimulate B cell proliferation and differentiation [116, 117].

B lymphocytes ultimately differentiate into two main types: short-lived plasmablasts and long-term plasma cells, both of which are able to produce antibodies. Plasmablasts are activated through T cell antigen presentation and initially differentiate in secondary lymphoid organs. They are short-lived, produce lower-affinity antibodies, and dominate the initial adaptive immune response [118]. In contrast, plasma cells also originate in secondary lymphoid organs but later migrate to supportive environments such as the spleen, lamina propria of the gut, and the bone marrow, where they further mature. Plasma cells have adapted metabolism and structural modifications that enable them to produce large quantities of high-affinity antibodies over extended periods. They are long-lived, rarely divide, and primarily produce IgG antibodies [119–121].

**Fig. 2:**
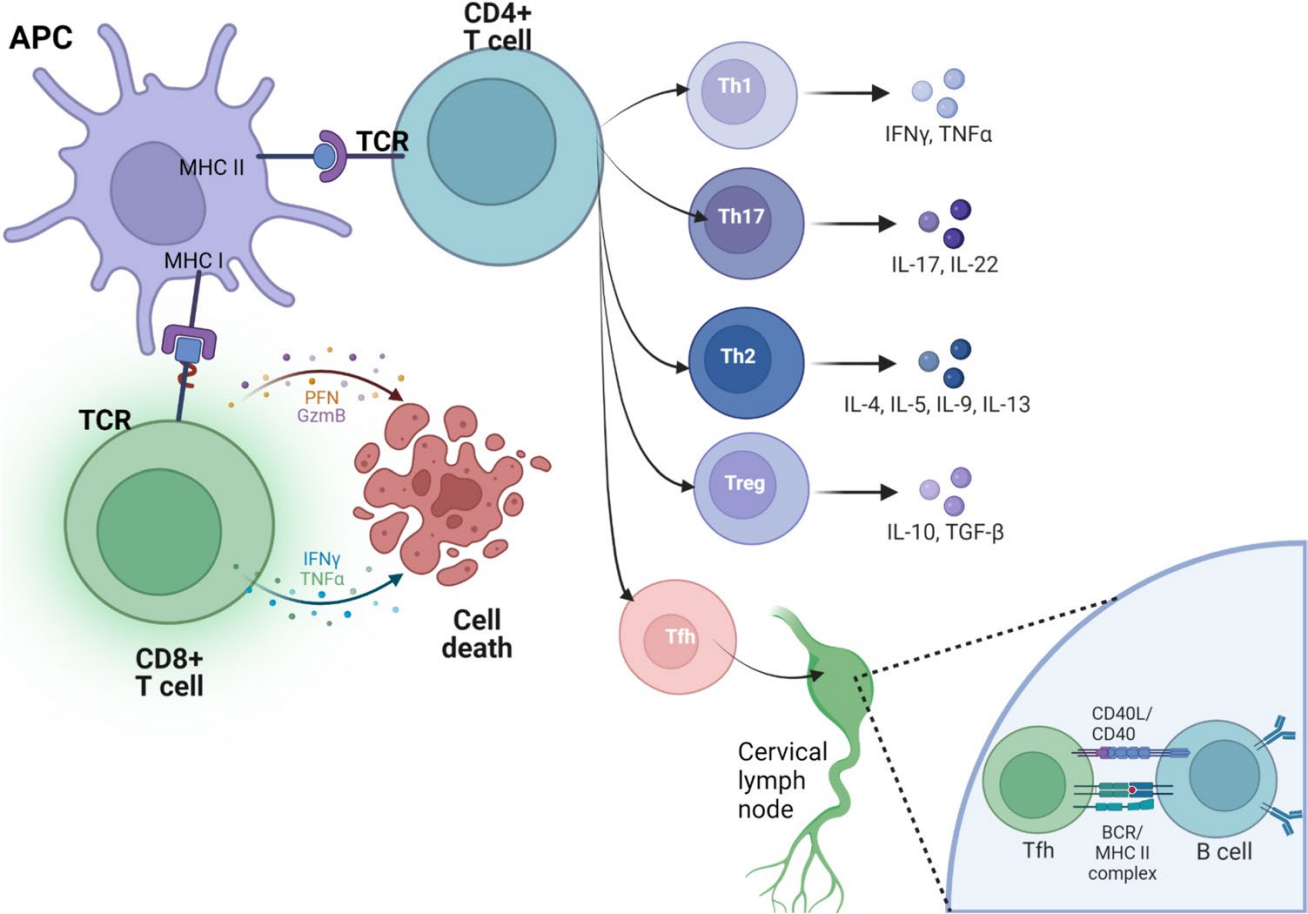
Immune Cell Recruitment and Differentiation in Response to Brain Injury. Recruitment of T lymphocytes into the injured tissue is facilitated by the upregulation of cell adhesion molecules on vascular endothelium. These adhesion molecules and chemokines are expressed in the leptomeninges and choroid plexus. Upon arrival at the site of injury, T cells come into contact with antigen-presenting cells (APCs), such as microglia and perivascular macrophages, which present antigen peptides to T cells through Major Histocompatibility Complex (MHC) Class I and II. MHC Class I is expressed on nearly all nucleated cells and presents endogenous peptides that are recognized by cytotoxic CD8 + T cells. These T cells produce pore-forming enzymes (PFN) and granzyme B (GzmB), inducing cytolysis and apoptosis through inflammatory mediators. In contrast, the MHC Class II/antigen complex is presented by APCs to naïve CD4 + T lymphocytes via the T cell receptor (TCR). MHC Class II antigen, in conjunction with other costimulatory molecules like CD80 and CD86, initiates a network of internal signaling that leads to the differentiation of naïve CD4 + T lymphocytes into five major types of CD4 + T helper (Th) cells: Th1, Th2, Th17, T regulatory (Treg), and T follicular helper (Tfh) cells. Th1 cells play a pro-inflammatory role by producing IL-2, TNF-α, and IFN-γ. Th17 cells also amplify inflammation through the production of IL-17, IL-22, and IL-23. Th2 cells produce anti-inflammatory or modulatory cytokines, including IL-4, IL-5, IL-10, and IL-13, and contribute to tissue repair by activating macrophages that induce fibroblast proliferation and the removal of extracellular matrix and apoptotic cells. Treg cells promote immune tolerance and attenuate T cell brain infiltration by expressing IL-10 and TGF-β. Tfh cells migrate to lymphoid tissue via the meningeal lymphatic system, initially reaching the cervical lymph nodes, where they present the MHC Class II/antigen complex to the B cell receptor (BCR) of B cells, thereby inducing the development of antigen-specific B cell immunity. T cells must display the CD40 ligand (CD40L), which binds to the CD40 receptor on the B cell surface as a costimulatory signal, leading to the maturation and activation of B cells. Acronyms: APC: Antigen-presenting cell; BCR: B cell receptor; CD40L: CD40 ligand; GzmB: Granzyme B; IFN-γ: Interferon-gamma; IL: Interleukin; MHC: Major Histocompatibility complex; PFN: Perforin; TCR: T cell receptor; Th: CD4 + T helper cell; Tfh: T follicular helper cell; Treg: T regulatory cell; TNF-α: Tumor necrosis factor-α; TGF-β: Transforming growth factor-β

After TBI, the initial humoral response is predominantly mediated by IgM-mediated, but later it is dominated by more antigen-specific IgG antibodies, which can persist for several months. These IgG antibodies may target myelin and neural components, and their persistence is associated with elevated neurofilament light concentrations, suggesting their contribution to ongoing neurodegeneration post-TBI [122] (Fig. 2). The release of brain proteins into the peripheral circulation, where they can function as antigens, has been documented. These proteins include glial fibrillary acidic protein (GFAP), myelin basic protein (MBP), neuron-specific enolase (NSE), glia calcium-binding protein S100B, ubiquitin carboxyl hydrolase like-1 (UCHL1) and neurofilament proteins among others [123, 124]. Levels of these biomarkers have been correlated with injury severity and prognosis [125]. Typically, these biomarker levels return to baseline within days or weeks after injury.

However, chronic auto-antibodies against these brain proteins have been documented for more than one year after trauma. Higher levels of these auto-antibodies have been associated with brain MRI abnormalities and cognitive changes [126–128]. Hypogonadotropic hypogonadism has been diagnosed in up to 36% of patients 12 months post-TBI and is positively correlated with the presence of IgM anti-pituitary and anti-hypothalamus autoantibodies, suggesting a role for autoimmunity in post-TBI hypopituitarism [122].

There is compelling evidence suggesting that TBI is a risk factor for neurodegenerative disorders such as Parkinson´s and Alzheimer´s disease [129]. TBI can cause axonal injury and disrupt microtubule function, which is one of the proposed mechanisms of amyloid-β and τ pathology, as well as protein misfolding, aggregation, and oxidative stress [130–132]. Post-traumatic proteinopathies share elements with neurodegenerative pathologies like Alzheimer´s and Parkinson´s. Additionally, BBB disruption and inflammatory response after TBI lead to microglia and astrocyte activation as well as infiltration by monocytes and lymphocytes. This persistent state of inflammation, which can last for years after trauma, can impair axonal growth and remyelination [133]. Reactive astrogliosis post-TBI is associated with changes in aquaporin-4 channel expression in the perivascular astrocytic extensions, disrupting the glymphatic system, reducing CSF flow, and impairing protein clearance, thereby contributing to protein accumulation and misfolding [134, 135].

In conclusion, TBI triggers both local inflammation in the brain and a systemic immune response through bidirectional communication between the CNS and the local and peripheral immune system. This inflammatory response initially aids tissue repair and vascular healing but, if unmodulated or excessive, can lead to harmful long-term effects. Although it is challenging to definitively establish a direct link between TBI and neurodegenerative disorders, there is growing evidence that dysregulated inflammation and autoimmunity post-TBI are related to cognitive and structural changes. During this mounting innate and adaptive immune response, the autonomic nervous system (ANS) plays a critical role in maintaining and regulating physiological homeostasis. The ANS also plays a key role in the surveillance and regulation of the inflammatory response [136, 137].

## Immune Response and its Association with the Autonomic Nervous System

A series of in vitro and in vivo studies have demonstrated that the cellular innate immune response is diminished in individuals who suffer a TBI [138]. Although the exact mechanisms remain unclear, several hypotheses have been proposed. One hypothesis involves DAMPs such as high-mobility group box 1 protein, interleukin-33, mitochondrial DNA, ATP, cytochrome C, HSPs, and S100 proteins. When released in high concentrations, these DAMPs trigger exaggerated inflammatory responses by activating specific receptors, including TLRs, C-type lectin receptors, NLRs, and absent in melanoma 2 (AIM2)-like receptors. These receptors are found in innate immune cells such as dendritic cells, granulocytes, microglia, and even non-immune cells like astrocytes, endothelial cells, and fibroblasts [139–141]. The interaction between these molecules causes further tissue injury, establishing vicious cycles of damage, which may lead to chronic inflammation [142].

Inflammatory cytokines produced in the acute phase of trauma, such as TNF-α, IL-1β, and IL-6, can interact with receptors outside the CNS on local afferent fibers of the vagus nerve or, after entering the bloodstream, with receptors in the CNS [143]. This creates bidirectional communication between the CNS and the immune system via the afferent sensory terminals of the vagus nerve. Inflammatory cytokines activate these terminals and direct a counter-regulatory response from the brain to restore immune homeostasis. This signaling occurs through three main pathways: the hypothalamic–pituitary–adrenal (HPA) axis, the sympathetic nervous system (SNS), and the parasympathetic nervous system (PNS) [144]. The HPA axis and the PNS generally have anti-inflammatory effects, while the SNS has been shown to regulate both innate and adaptive immunity. An imbalance in these systems can exacerbate inflammatory responses, complicating the clinical picture in TBI patients [145]. Understanding these interactions is crucial for developing therapeutic strategies to modulate the immune response and improve outcomes in TBI.

## Sympathetic Response to Acute Brain Injury

The SNS plays a crucial role in modulating the immune response, as evidenced by the presence of adrenergic receptors on nearly all leukocytes and in both primary (bone marrow, thymus) and secondary (spleen, lymph nodes, gut-associated lymphoid tissue) lymphoid organs [146]. Catecholamines, produced in response to inflammatory and stress-mediated activation of the autonomic system and subsequent stimulation of the HPA axis, can suppress immunity through β2-adrenergic receptors on dendritic cells. Stimulation of the β2-adrenergic receptor reduces TNF-α production [147]. This suppression indirectly reduces the production of cytokines such as IL-12, which is essential for the differentiation of helper T cells into type 1 helper T cells (Th1) responsible for producing IFN-γ and activating CD8 + T cells and natural killer cells [148, 149].

An animal model study by Prass et al. demonstrated that inhibiting β2-adrenergic receptors in mice with ischemic stroke significantly reduced bacterial infection rates and increased survival by preventing interferon-γ production induced by the sympathetic system counter-regulatory response to the ischemic event and subsequent inflammation [150].

Furthermore, the SNS inhibits pro-inflammatory cytokine production while enhancing anti-inflammatory cytokines like IL-10 [151, 152]. A novel mechanism, T cell exhaustion, has been studied closely, revealing that the sympathetic response and norepinephrine release induce CD4 + and CD8 + T cell functional impairment by upregulating programmed cell death 1 (PD-1) expression after β2 receptor stimulation. This impairment was effectively reversed in vivo and in vitro by administering the β-blocker propranolol [153]. Understanding these interactions is crucial for developing therapeutic strategies to modulate the immune response and improve outcomes in patients with acute brain injury [154].

## Parasympathetic Response to Acute Brain Injury

The PNS, often referred to as the "rest and digest" counterpart to the SNS, also plays a crucial role in the body's response to acute brain injury [155]. NE is involved in an additional mechanism of the ANS, specifically activating the cholinergic anti-inflammatory pathway [155, 156]. NE released by a sympathetically stimulated splenic nerve binds to adrenoreceptors in splenic T cells, provoking acetylcholine (ACh) release. This ACh then binds to α7 nicotinic acetylcholine receptors (α7nAChRs) on splenic leukocytes, leading to polarization of cytokine production toward anti-inflammatory mediators from these leukocytes [157, 158].

**Fig. 3:**
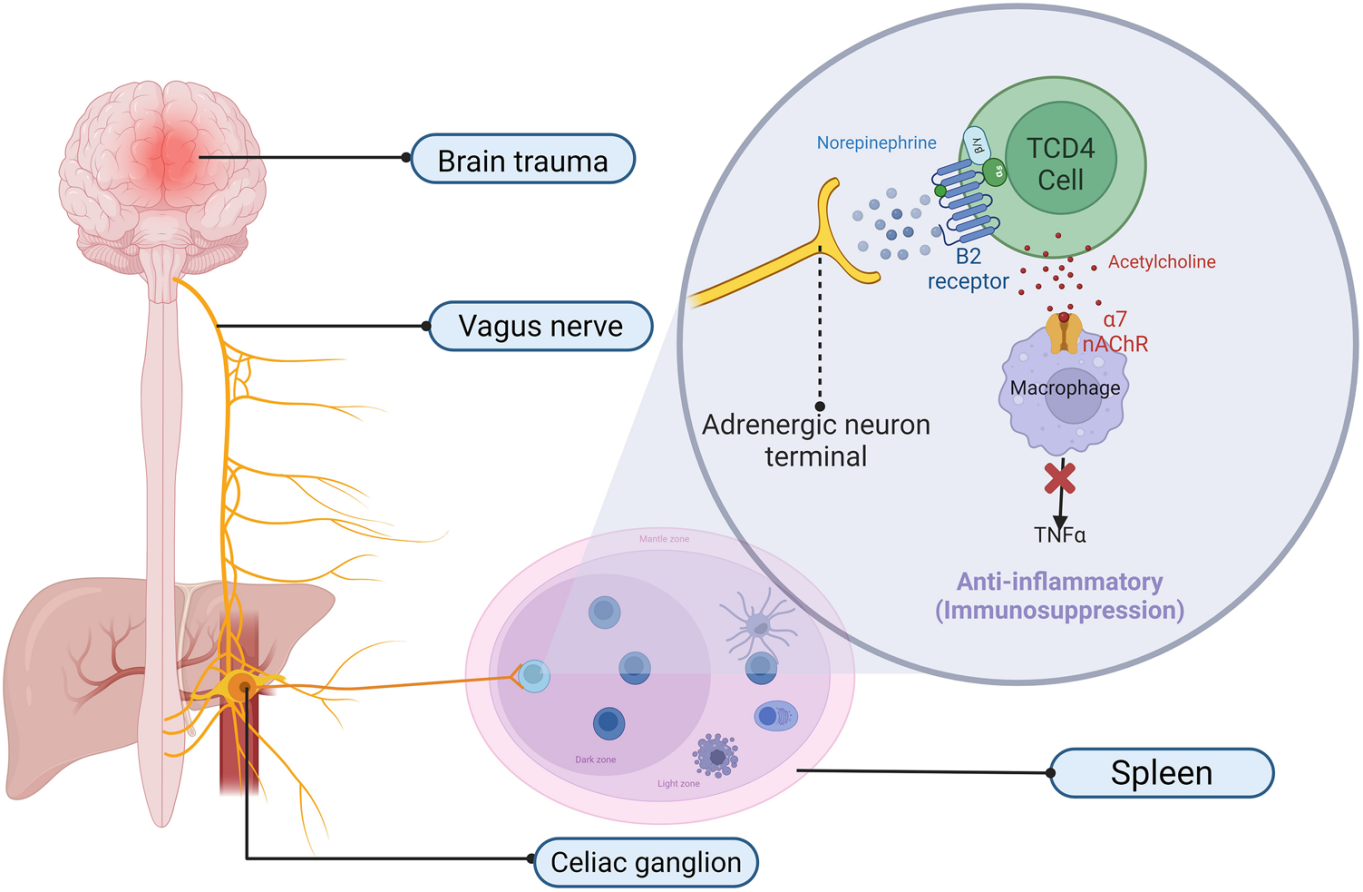
The Neuroimmune Reflex in Response to Trauma. Activation of the vagus nerve by inflammatory cytokines following trauma triggers a reflex response involving the celiac ganglion. This ganglion, which is sympathetically connected to the spleen, stimulates the release of norepinephrine. Norepinephrine activates β2-adrenergic receptors on splenic CD4 + T lymphocytes, which in turn produce acetylcholine (ACh). ACh further stimulates splenic leukocytes, such as macrophages and dendritic cells, via the α7 nicotinic acetylcholine receptor (α7nAChR), resulting in a reduction of pro-inflammatory cytokines like TNF-α. Acronyms: ACh: Acetylcholine; α7nAChR: α7 Nicotinic Acetylcholine Receptor; TNF-α: Tumor Necrosis Factor-α

This anti-inflammatory response is further supported by stimulating numerous vagus nerve endings, triggering a reflex response in the nucleus tractus solitarius (NTS) via release of ACh in various organs [159]. These findings suggest acute brain injury enhances parasympathetic activity in response to initial sympathetic stimulation. There is also a reflexive response from the vagus nerve to the initial release of ACh by splenic leukocytes, as well as the direct stimulation of the NTS. These pathways may contribute, at least in part, to the peripheral immune suppression observed following TBI [160, 161]. If further validated, this theory suggests that modulating vagus nerve activity could offer a novel therapeutic approach to reducing not only the direct mortality associated with infection but also the long-term neurological consequences of infection in patients with TBI and acute brain injury [162, 163] (Fig. 3).

## Conclusion

The immune response is an essential element in the pathophysiology of TBI, significantly contributing to the development of secondary injury. Initially, cell injury causes the release of DAMPs with an affinity for PRR, triggering the assembly of inflammasomes and other intracellular pathways involved in complement activation and cell death and survival processes.

Inflammasomes are protein complexes that activate caspases, inducing the activation of IL-1β and triggering programmed cell death pathways such as pyroptosis. This initial response is carried out by the innate immune system, the body’s first line of defense against injury and infection. Innate immunity is characterized by rapid response, and the ability to recognize a broad range of danger signals through PRRs within minutes to hours following the initial insult. Subsequently, the adaptive system is involved and initiates a response characterized by specificity and memory, allowing for a more tailored response to the injury.

Traditionally, the CNS was considered an immune-privileged site. However, TBI can disrupt the BBB, allowing peripheral immune cells to infiltrate the brain and participate in this adaptive immune response. The ANS is part of the link between the peripheral immune system and the CNS, and it is extensively activated in situations of metabolic distress, such as acute brain injury. Peripheral lymphoid organs have receptors for ACh and NE, and the ANS influences their activity. Autonomic stimulation can contribute to a state described as CNS-induced immune deficiency syndrome, where immune exhaustion and suppression impair the body's ability to respond to infections.

The balance between pro-inflammatory and anti-inflammatory responses is critical in determining the extent of secondary injury and the potential for recovery. Advances in understanding these mechanisms offer promising avenues for therapeutic interventions aimed at mitigating the detrimental effects of neuroinflammation and promoting neuroprotection and repair in TBI patients. Emerging therapeutic strategies are showing promise in targeting the immune system in TBI. Anti-inflammatory drugs, including corticosteroids, aim to reduce neuroinflammation and secondary injury, though their efficacy has been variable. Biologics, such as monoclonal antibodies targeting specific inflammatory cytokines like IL-1β and TNF-α, are being explored for their potential to modulate the immune response more precisely. Additionally, immune modulation techniques, such as vagus nerve stimulation (VNS) and β-blockers, are under investigation for their ability to influence the autonomic response and immune system. Regenerative medicine approaches, including stem cell therapies and tissue engineering, are also being studied for their potential to repair damaged brain tissue and modulate immune responses.

Despite these advancements, current therapies face limitations, including modest efficacy and potential adverse effects. The complexity of the immune response in TBI, with its interplay between innate and adaptive systems, underscores the need for further research to fully understand these mechanisms and develop more effective treatments. Future research should focus on identifying reliable biomarkers for monitoring immune responses, understanding the precise mechanisms of immune-related secondary injury, and conducting robust clinical trials to evaluate new therapies.

Incorporating patient perspectives is crucial, as effective immune modulation can significantly impact patient outcomes and quality of life. TBI patients often face long-term cognitive, emotional, and physical challenges, and improving recovery through better immune modulation can enhance functional outcomes and overall quality of life. Addressing these research needs and integrating patient feedback aims to mitigate neuroinflammation, promote neuroprotection, and improve recovery for individuals affected by TBI.

## Data Availability

Data that support the findings of this study have been collected in REDCap supported by Universidad de La Sabana.

## Abbreviations

α7nAChRs: Alpha-7 nicotinic acetylcholine receptors
Ach: Acetylcholine
ALR: AIM2-like receptors
AN: Autonomic Nervous
APC: Antigen presenting cell MHC major histocompatibility complex
BBB: Blood brain barrier
BCR: B cell receptor
CARD: Caspase activation and recruitment domain
CD: Cluster of differentiation
CLR: C-type lectin receptors
CNS: Central nervous system
CSF: Cerebrospinal fluid
DAMP: Damage associated molecular pattern
ECAM-1: Endothelial cell adhesion molecule 1
GFAP: Glial fibrillary acidic protein
GOSE: Glasgow Outcome Scale-Extended
GSDMD: Gasdermin D
GzmB: Granzyme B
HPA: Hypothalamic-pituitary-adrenal
HMGB1: High mobility group box-1
HSP: Heat shock protein
ICAM-1: Intercellular adhesion molecule 1
IFN: Interferon
IFN-γ: Interferon gamma
IFNg: Interferon-g
IRF: Interferon regulatory factor
MAC: Membrane attack complex
MAPKs: Mitogen-activated protein kinases
MASP: Mannose-binding lectin associated serine proteases
MBL: Mannose binding lectin
MDA5: Melanoma differentiation-associated gene-5
NF-κB: Nuclear factor kappa-light-chain-enhancer of activated B cells
NLR: Nod-like receptor
NLRP: Nod-like receptor protein
NSE: Neuron-specific enolase
NTS: Nucleus tractus solitarius
PFN: Perforin
PNS: Parasympathetic nervous system
PRR: Pattern recognition receptors
RIGig-1: Retinoic acid-inducible gene-1
RLR: Rig-like receptor
ROS: Reactive oxygen species
SNS: Sympathetic nervous system
SR: Scavenger receptor
TBI: Traumatic brain injury
TCR: T cell receptor
Th: T helper
TNF-α: Tumor necrosis factor alpha
TLR: Toll-like receptor
UCHL1: Ubiquitin carboxyl hydrolase like-1
VCAM-1: Vascular cell adhesion molecule 1
VNS: Vagus nerve stimulation
